# Low barrier buprenorphine treatment for persons experiencing homelessness and injecting heroin in San Francisco

**DOI:** 10.1186/s13722-019-0149-1

**Published:** 2019-05-06

**Authors:** Jamie Carter, Barry Zevin, Paula J. Lum

**Affiliations:** 1 Lincoln Community Health Center, 1301 Fayetteville St, Durham, NC 27707 USA; 2 Street Medicine and Shelter Health, San Francisco Department of Public Health, 101 Grove St, San Francisco, CA 94102 USA; 3 UCSF Division of HIV, Infectious Disease and Global Medicine, Zuckerberg San Francisco General, 1001 Potrero Ave, San Francisco, CA 94110 USA

**Keywords:** Opioid use disorder, Low barrier buprenorphine, Buprenorphine, Homelessness

## Abstract

**Background:**

Opioid overdose is a leading cause of death in persons experiencing homelessness (PEH), despite effective medications for opioid use disorder (OUD). In 2016, the San Francisco Street Medicine Team piloted a low barrier buprenorphine program with the primary goal of engaging and retaining PEH with OUD in care as a first step toward reducing opioid use and improving overall health.

**Objective:**

To characterize the patients; assess treatment retention, retention on buprenorphine, and opioid use; and to describe adverse events.

**Methods:**

Retrospective chart review of patients receiving at least one buprenorphine prescription from Street Medicine (November 2016–October 2017). We abstracted demographic, medical, substance use, prescription, and health care utilization data from medical records. We assessed retention in care at 1, 3, 6, 9 and 12 months, defined as a provider visit 1 week prior to or any time after each time point. We considered patients to be retained on buprenorphine if they had active buprenorphine prescriptions for more than 2 weeks of the month. We estimated opioid use by the percentage of patients with any opioid-negative, buprenorphine-positive urine toxicology test. We reviewed emergency department and hospital records for adverse events, including deaths and nonfatal opioid overdoses.

**Results:**

Among the 95 persons eligible for analysis, mean age was 39.2, and 100% reported injecting heroin and homelessness. Medical and psychiatric comorbidities and co-occurring substance use were common. The percentages of patients retained in care at 1, 3, 6, 9 and 12 months were 63%, 53%, 44%, 38%, and 26%, respectively. The percentages of patients retained on buprenorphine at 1, 3, 6, 9 and 12 months were 37%, 27%, 27%, 26%, and 18%, respectively. Twenty-three percent of patients had at least one opioid-negative, buprenorphine-positive test result. One patient died from fentanyl overdose, and four patients presented on six occasions for non-fatal overdoses requiring naloxone.

**Conclusions:**

This program engaged and retained a subset of PEH with OUD in care and on buprenorphine over 12 months. While uninterrupted treatment and abstinence are reasonable outcomes for conventional treatment programs, intermittent treatment with buprenorphine and decreased opioid use were more common in this pilot and may confer important reductions in opioid and injection-related harms.

## Background

Despite the existence of evidence-based treatment for opioid use disorder (OUD), opioid overdose deaths continue to rise [1]. Only one in five people with OUD in the United States receives any treatment [2], and only 37% of people receiving treatment are prescribed the medications buprenorphine or methadone [3, 4], which improve health outcomes and reduce mortality [5–9]. This treatment gap highlights the need for interventions that address barriers to care and engage out-of-treatment people who use drugs. Barriers that prevent people from receiving appropriate OUD treatment include availability, cost and stigma [10–12].

Persons experiencing homelessness (PEH) have higher rates of substance use disorders [13] and substance-related mortality than the general population [14], with opioid overdose as a leading cause of death [15]. Buprenorphine is a recommended treatment for OUD among PEH [16], but these patients face additional barriers to care, including social isolation, discrimination, and competing priorities [16–19].

Models of care that lower thresholds to treatment with methadone have been developed in Canada and Europe, with success in retaining marginalized patients in care [20, 21], reducing overdose and all-cause mortality [22, 23], and decreasing injection-related risk behaviors among patients who continue to use drugs [24]. While the model is not fully developed and there is some variability in practice, “low threshold” methadone programs prioritize the reduction of drug-related harms over abstinence as the primary treatment goal. Such programs feature flexible attendance and urine drug testing requirements and do not discharge patients with ongoing drug use [25].

Harm reduction syringe access programs are a major provider of services to out-of-treatment people with OUD, and people using these services are often homeless and have faced barriers to accessing buprenorphine treatment despite interest [26–30]. Past interventions to reach these marginalized patients include buprenorphine treatment linkage through harm reduction staff education, motivational interviewing, and referral training [31], and pilot programs that directly provided buprenorphine treatment within harm reduction agencies [32–34]. One such pilot program in New York City provided immediate clinical assessment and same-day prescription for buprenorphine, did not require counseling or urine toxicology testing, and accepted patients with goals other than abstinence. Patient retention in the program was 68%, 63%, 56%, and 42% at the end of 3, 6, 9 and 12 months, respectively [32].

Other models of lower threshold treatment for PEH in the United States include buprenorphine treatment provided by a multidisciplinary team at family shelters in Boston, which found that shelter-based treatment was feasible and may have helped patients decrease opioid use, avoid overdose, and maintain employment [35]. Another study found that mobile methadone vans in New Jersey were able to engage a greater proportion of non-white, homeless, uninsured people who inject drugs (PWID) compared to traditional methadone clinics [36].

In 2016, the San Francisco Street Medicine Team started a low barrier buprenorphine treatment pilot program after identifying a need for more accessible treatment among PEH who use heroin. There are an estimated 22,500 people who inject drugs in San Francisco, many of whom are experiencing homelessness, and heroin use and fentanyl deaths are increasing [37].

The Street Medicine Team cares for PEH who are not otherwise able to get their health needs met within San Francisco’s relatively robust safety-net system of care. Patients are engaged by peer outreach workers or self-present on a drop-in basis to either a small open-access medical clinic or a nearby syringe access program, where a clinician provides comprehensive substance use assessment and education and calls in a same-day prescription for buprenorphine/naloxone to be filled at a community pharmacy that dispenses the medication free to patients who are uninsured or have Medicaid. This pharmacy is operated by the Department of Public Health and provides medications for mental health and substance use disorders to the city’s safety net population. In 2018, the Street Medicine Team also began providing treatment at local shelters and homeless encampment health fairs, and some patients who were initially engaged in the clinic or at the syringe access program continued their treatment through these sites. Any patient with OUD experiencing homelessness who is interested in buprenorphine treatment is eligible to participate, including patients with alcohol and benzodiazepine use disorders, pregnant patients, and youth.

Clinicians determine specifics of patient care plans in a flexible manner with attention to prior barriers faced and with support for patients who have ongoing substance use, goals other than abstinence, and treatment interruptions. The team provides an initial prescription for 3–7 days of buprenorphine/naloxone, and patients have weekly visits early in treatment. With written instructions, patients manage their own “home” induction at the location of their choice and are able to titrate to a typical initial dose of 16 mg. Urine toxicology testing is typically performed at least monthly, with testing done more frequently if there are clinical indications. In some cases toxicology testing may be a barrier to care and is deferred, for example if there is no place to collect a sample or if the patient has had prior traumatic experiences with urine testing and would opt to forgo treatment rather than complete the test. Depending on the treatment site, toxicology testing is either point-of-care or send-off testing to the local hospital laboratory.

As they progress in treatment, patients who are stable with abstinence from opioids may have visits as little as monthly. Patients who continue to use heroin but have improvement in functioning and are satisfied with their treatment are not considered unstable and are typically seen every 1–2 weeks. In cases of clinical instability, the team focuses on keeping the patient engaged in care, strongly encouraging higher levels of care when appropriate but also recognizing that realistically many patients will face barriers to engagement in higher levels of care. Patients may be offered the choice of ongoing care through the Street Medicine team with daily observed buprenorphine dosing on weekdays through the community pharmacy or transition to methadone maintenance or residential treatment. Counseling is available and encouraged through partnership with the Center for Harm Reduction Therapy but is not required.

The primary goal of the pilot program is retention in care, with secondary goals of improved health, reduction in opioid use, and abstinence. The Street Medicine team’s target population is highly marginalized and mistrustful of the medical community with a substantial burden of chronic physical and mental illness, and thus the development of trust to facilitate ongoing engagement for medical care, mental health care, harm reduction services, and case management is valuable even in patients who are not continuing to take buprenorphine.

This study aims to describe the results of the pilot program by characterizing the population participating in low barrier buprenorphine treatment, assessing retention in treatment, retention on buprenorphine and reduction in opioid use, and reporting adverse events.

## Methods

The study population consisted of all patients who had at least one visit with the Street Medicine Team for buprenorphine treatment between November 1, 2016 and October 31, 2017. We abstracted data on gender, age, and race/ethnicity from the electronic medical record’s demographic section. We collected data on medical and psychiatric comorbidities, and use of opioids, cocaine, methamphetamines, benzodiazepines, and alcohol, from Street Medicine provider notes at the time of initial visit, the active problem list, and diagnoses in other provider visit notes from the year prior to admission to the program if Street Medicine provider notes were not available. Data on substance use at the time of initial visit was based on patient self-report. We defined unhealthy alcohol use to be use that exceeds NIAAA thresholds for risky drinking [38] or use in any patient with a diagnosis of alcohol use disorder.

We collected data on the following outcomes from the time of initial patient visit through October 31, 2018, such that all patients in the cohort had 12 months of follow-up. We abstracted data on frequency of Street Medicine provider visits from the medical record. We reviewed prescription drug monitoring program data for each patient as part of patient care and quality improvement efforts and used this to determine patterns of active buprenorphine prescription among participants. We collected results from all urine toxicology and urine buprenorphine tests that were completed after the initial visit.

We evaluated retention in care, the primary outcome, at 1, 3, 6, 9 and 12 months. We defined a patient to be retained in care at a particular time point if they had any visit with a Street Medicine provider within 1 week prior to or at any time after the time point in question. As a secondary measure, we also determined whether the patient had any visit with a provider during the month in question, where a follow-up visit in the first month had to be in addition to the initial visit. We used this second measure because some patients had extended absences before eventually returning to care, which are not captured with the first measure of retention.

Some patients appropriately transitioned to methadone maintenance or office-based opioid treatment through traditional primary care clinics during the study period. When the research team was aware of these transitions, these patients were considered to be retained in care while receiving treatment through the new provider.

We defined a patient to be retained on buprenorphine during the month in question if they had an active prescription for buprenorphine for more than 2 weeks of the 4-week period. Patients did not have to have active prescriptions in the prior months to be considered retained on buprenorphine during the month in question if they met these criteria. We defined a patient’s maintenance buprenorphine dose to be the maximum dose taken for at least 2 continuous weeks.

Urine toxicology testing was performed in a non-standardized way based on provider discretion and clinical circumstances, and thus patients had different numbers of tests at different time-points. To highlight this, we calculated the mean, median and range for the number of toxicology tests performed. We report the percentage of all toxicology tests done in the cohort that were positive for opioids, methamphetamines, cocaine, benzodiazepines, and buprenorphine. We are not able to report or compare toxicology test results among individual participants at specific time-points because of the variability in testing practices. We report the percentage of patients who had any toxicology test that was negative for non-prescribed opioids and positive for buprenorphine.

We assessed adverse events by reviewing emergency department and hospital admission records for all San Francisco hospitals during participants’ periods of retention in care. One physician reviewed any ED visit or hospital admission note to determine whether the encounter was potentially related to buprenorphine treatment, including opioid overdose, precipitated withdrawal or other adverse effects. In the case of overdose, the physician reviewed available documentation and test results to determine whether treating providers suspected that buprenorphine was a cause, or whether use of alcohol or benzodiazepines concurrent with buprenorphine may have been related. The San Francisco medical examiner notifies the Street Medicine medical director of any deaths among PEH in the city, so the team was made aware of any deaths that occurred in the cohort.

## Results

Ninety-five patients had at least one visit for buprenorphine treatment with the Street Medicine team and were included in the study. Mean age was 39.2, and most patients were male (74%) and white (68%). Fifty-eight percent of patients had a chronic medical condition, such as hypertension or chronic hepatitis C infection, and 66% had a psychiatric condition, including 26% with bipolar disorder or a psychotic disorder. All participants used heroin and engaged in injection drug use. At baseline, 61% used methamphetamines, 26% used cocaine, 8% used benzodiazepines, and 12% met criteria for unhealthy alcohol use (see Table 1). Twenty-four percent of patients had previously sought treatment at the San Francisco Office-Based Buprenorphine Induction Clinic.

**Table 1:** Demographic and clinical characteristics of participants

Demographic and clinical characteristics	Total N = 95 N (%)
Age *mean* (range)	39.2 (22–66)
Male	70 (74%)
Female	25 (36%)
Race	
White	65 (68%)
Black	17 (18%)
Hispanic	8 (8%)
Other	5 (5%)
Chronic medical condition	55 (58%)
Psychiatric condition	63 (66%)
Bipolar or psychotic disorder	25 (26%)
Baseline substance use	
Heroin	95 (100%)
Methamphetamines	59 (61%)
Cocaine	25 (26%)
Benzodiazepines	8 (8%)
Unhealthy alcohol use	11 (12%)
Injection drug use	95 (100%)

**Fig. 1:**
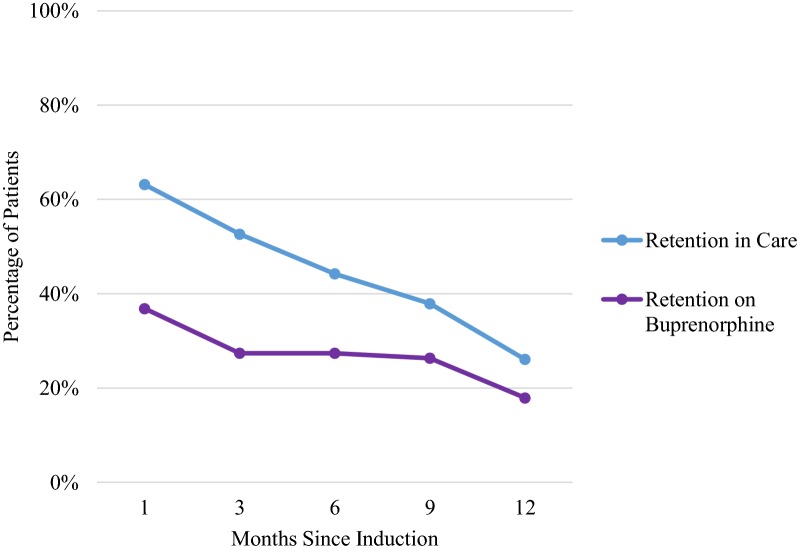
Retention in care and on buprenorphine by month

The majority (64%) of initial clinical assessments took place in the open-access medical clinic, with some occurring at the syringe access program (29%) and on the street (8%). Seventy-four percent of patients returned for follow-up after the initial visit at least once during the 12 months of evaluation. Sixty-three percent of patients were retained in care at 1 month, 53% at 3 months, 44% at 6 months, 38% at 9 months, and 26% at 12 months (Fig. 1). The percentages of patients who had a follow-up visit in the first, 3rd, 6th, 9th, and 12th months, were 55%, 41%, 38%, 34%, and 26%, respectively. Interruptions in treatment were common: among patients who followed-up after intake, 46% had a treatment interruption of 1 month or longer with subsequent return to care.

The percentages of patients retained on buprenorphine at 1, 3, 6, 9 and 12 months were 37%, 27%, 27%, 26%, and 18%, respectively (Fig. 1). The average maintenance buprenorphine dose was 20.9 mg.

Twenty-nine patients were retained on buprenorphine for at least two of the evaluation time points (months 1, 3, 6, 9, or 12). Among these patients, 14 (48%) had continuous active prescriptions for buprenorphine during the time they were treated. Five (17%) of these patients had an interruption in their buprenorphine prescription of 2–3 weeks, 8 (28%) had an interruption of 4–6 weeks, and 5 (17%) had an interruption of greater than 6 weeks. Seven patients (24%) had multiple interruptions.

Two hundred and six urine toxicology tests were completed by the cohort, and 71% of patients who followed up after intake had a toxicology test, with a mean of 2.7 tests and a median of one test per follow-up patient (range 0–25). Sixty-three percent of urine toxicology tests were positive for opioids, 73% for methamphetamines, 25% for cocaine, 10% for benzodiazepines, and 81% for buprenorphine. Twenty-three percent of patients had at least one opioid-negative, buprenorphine-positive toxicology test.

One patient in the cohort, who was not recently engaged with the Street Medicine team or actively on buprenorphine maintenance, died from fentanyl and methamphetamine overdose. Four patients received emergency or inpatient medical treatment for an opioid overdose requiring naloxone, and one of these patients had three overdoses that required naloxone, for a total of six overdoses requiring naloxone in the cohort. The patient with three overdoses had active buprenorphine prescriptions at the times of overdose, as did two of the other three patients with overdose. One patient who had an overdose requiring naloxone also used benzodiazepines, though his overdose was during an admission to a medically supervised withdrawal facility just prior to buprenorphine induction. Five patients were treated for possible opioid overdose events not requiring naloxone, with two of five having active buprenorphine prescriptions at the times of possible overdose. One of the patients with a possible overdose event also used benzodiazepines. None of the patients with confirmed or possible overdose events had known unhealthy alcohol use. None of the overdose events were thought by the treating physicians to be caused by buprenorphine: in all cases documentation reflected lack of awareness that the patient was in treatment with buprenorphine, and the overdose was thought to be due to heroin or fentanyl.

## Discussion

In this retrospective descriptive chart review study, we found that a low barrier buprenorphine pilot program for PEH with OUD was successful in engaging and retaining a subset of patients in care and in continued treatment with buprenorphine.

While our rates of retention in care, ranging from 53% at 3 months to 26% at 12 months, are lower than those found in conventional office-based buprenorphine treatment programs, our results are promising because of our high-risk population of chronically homeless patients not able to access care in other settings, many of whom are not treatment-seeking at the time of engagement. San Francisco’s Office-Based Buprenorphine Induction Clinic’s program reported retention in care of 61% at 1 year among a treatment-seeking population that excluded many patients with severe mental illness and other substance use disorders, only 40% of whom were homeless [39]. A study that compared outcomes of housed and homeless patients in a primary care-based buprenorphine treatment program found exceptionally high retention of almost 90% at 1 year in both groups, likely explained by the fact that patients had to “demonstrate appropriateness” during an initial treatment period including adherence to medical visits, mental health stability and lack of other substance use disorders [40]. In the first reported study of buprenorphine treatment delivered at a harm reduction agency in New York City (2012), retention was 63% at 6 months and 42% at 12 months, likely higher than our results again because of differences in the population: almost half of patients were employed, and only 50% engaged in injection drug use. Retention estimates included only patients who returned after the initial visit, whereas our analysis includes all patients with at least one visit with the program [32].

Over a quarter of patients were retained on buprenorphine at 6 months, and nearly one-fifth were retained at 1 year. Our results of 27% buprenorphine treatment retention at 6 months are comparable to those of Stancliff et al., who found that 31% of patients treated through a harm reduction agency were continuously maintained on buprenorphine over 6 months [32]. Interruptions in treatment were common in our population, likely reflecting the chaotic lives and competing priorities of chronically homeless adults with active substance use. To capture patients who had treatment interruptions but also kept returning to the program over a significant period of time, we defined a patient to be retained on buprenorphine during a particular month if they had more than 2 weeks of active buprenorphine prescription during the month. Because most buprenorphine prescriptions were written on a weekly basis for patients with less stability and for those in the earlier stages of treatment, patients meeting this threshold typically had at least three active weeks of buprenorphine prescription during the month. Some patients appropriately transitioned to methadone maintenance during the study period which may explain some of the decrease in buprenorphine retention over time, and others may have had interruptions in treatment due to incarceration and hospitalization.

While continuous maintenance treatment with buprenorphine is the standard of care in conventional programs, intermittent treatment with buprenorphine was common in our population and may still provide benefits. First, pharmacologic studies show that even single moderate doses of buprenorphine result in nearly complete attenuation of the effects of opioids up to 72 h after a dose [41, 42], and thus intermittent buprenorphine use may be protective against opioid overdose during periods of use. With fentanyl contaminating the drug supply, any single dose of buprenorphine taken may be life-saving. A study of receipt of medication for opioid use disorder after nonfatal overdose found that as little as 1 month of treatment with buprenorphine over 1 year was associated with reduced mortality, suggesting that intermittent or short-term use of buprenorphine can be beneficial [43]. Another study found an incremental benefit of increasing levels of adherence to buprenorphine treatment for preventing return to use and decreasing acute health care utilization [44]. Exploration of the potential benefits of intermittent use of buprenorphine is an area that deserves further study.

Our urine toxicology results reflect adherence to buprenorphine concurrent with ongoing use of heroin and methamphetamines in a majority of the cohort. We found some evidence of periods of opioid abstinence, with 23% of patients having at least one opioid-negative, buprenorphine-positive test. In our clinical experience, many patients report taking buprenorphine regularly and using substantially less heroin, while still using heroin occasionally. We are exploring this phenomenon further through qualitative research and in-depth interviews with participants, as it is difficult to measure a decrease in amount of heroin use with the binary tool of a urine toxicology test.

When considering how best to approach treatment for patients with ongoing use of other substances, two studies found retention in buprenorphine treatment equivalent among patients who did and did not use cocaine [45, 46], with stimulant use also declining during buprenorphine treatment in one study [46]. In our population, use of methamphetamines likely destabilizes many patients and may contribute to worse treatment outcomes, but denying these patients care for their opioid use disorder because of their stimulant use risks causing further harm. Some of our patients have unhealthy alcohol (12%) and benzodiazepine use (8%), which in the past have been considered contraindications to treatment with buprenorphine. Our approach to these patients aligns with recent guidance from the FDA that recognizes the substantial mortality risk from untreated opioid use disorder and recommends careful treatment with buprenorphine and methadone in patients with comorbid sedative use that can reduce overall risks [47].

Adverse events, including one death and multiple overdose events requiring naloxone, likely reflect the severity of opioid use disorder in the population and high mortality of this condition, rather than adverse events caused by buprenorphine treatment itself. Though review of the medical record may not provide a complete picture, we found no evidence to suggest that the overdose events were buprenorphine overdoses, rather than overdoses on heroin and fentanyl consistent with the ongoing high rates of opioid use in the population.

The ability of this buprenorphine pilot program to retain in care persons with OUD experiencing homelessness may be due to a number of low-barrier characteristics. These include engagement by peer outreach workers, flexible, drop-in treatment availability, provision of treatment at a syringe access program where many out-of-treatment people are already accessing services regularly, same-day prescriptions for buprenorphine, collaboration with a pharmacy that can dispense medication to patients without insurance, optional substance use counseling, and continued treatment for patients who have treatment interruptions and ongoing substance use. Though a precise model for “low barrier” buprenorphine treatment has not been formally developed, evidence from other settings support the tenets of such a program.

First, methadone maintenance programs centered around a harm reduction philosophy which reject abstinence as a required goal of treatment have been shown to retain vulnerable patients in care [20, 21], reduce mortality [22, 23], and decrease injection-related risk behaviors [24]. Provision of treatment at syringe access programs is preferred by out-of-treatment PWID, particularly those who have faced barriers to care [26–30]. Requirements for additional counseling beyond brief medication management during the medical visit can be a treatment barrier for persons with already low engagement in care. Adjunctive psychosocial interventions, moreover, have been shown in some addiction pharmacotherapy studies to confer no additional benefit over medication management [48, 49].

A study of rapid, same-day intake into a methadone maintenance program that increased retention substantially over 5 months compared to usual care [50] supports the idea that treatment on-demand improves retention in care. Our collaboration with a pharmacy that shares our harm reduction philosophy and dispenses buprenorphine to patients without insurance likely contributes to our retention in care by facilitating same-day buprenorphine initiation. The city of San Francisco funds buprenorphine costs for patients without insurance, with a program goal to help the patient obtain insurance after stabilization on buprenorphine. Requirements for photo identification are often barriers to same-day treatment, particularly for PEH who may not have a valid ID. Some states allow pharmacist discretion to dispense controlled substances if refusing to do so because of lack of identification would be a detriment to the patient [51]. If more states adopted this approach, access would improve for vulnerable populations.

This study has several limitations. This was a retrospective analysis of chart review data with no comparison or control group, and thus we are not able to draw conclusions about how outcomes of our low barrier approach compare to those of other approaches to treatment in this population. The quality of demographic data in the electronic medical record was poor, with conflation of race and ethnicity and lack of information on patients identifying as transgender. Frequency of urine toxicology testing varied among participants, so results could be skewed by participants who had more tests and may not be an accurate reflection of the cohort’s substance use. We are not able to report or compare toxicology test results among individual participants at specific time-points because of the variability in testing practices. We are not able to assess buprenorphine adherence apart from reporting the overall percentage of buprenorphine positive toxicology tests. We describe what happened in a real-world pilot program where aspects of the intervention varied among participants based on providers’ clinical judgment.

## Conclusions

In conclusion, this study found that a low barrier buprenorphine pilot program successfully engaged and retained a subset of marginalized PEH in care and in continued treatment with buprenorphine. While continuous treatment with buprenorphine and opioid abstinence may be feasible and achievable goals of conventional OUD treatment programs, intermittent treatment with buprenorphine and decreased opioid use were more common in this pilot and may confer significant reductions in opioid and injection-related harms. To decrease the OUD treatment gap and engage marginalized populations, buprenorphine treatment programs should consider how traditional treatment models can be disrupted to increase exposure to effective medication, reduce any amount of opioid use, and eliminate barriers to more accessible care.

## Data Availability

The datasets used and/or analyzed during this study are available from the corresponding author upon reasonable request.

## Abbreviations

OUD: opioid use disorder
PWID: people who inject drugs
PEH: persons experiencing homelessness
